# 17β-Hy­droxy-17α-methyl­androsta-1,4-dien-3-one

**DOI:** 10.1107/S1600536812049860

**Published:** 2012-12-08

**Authors:** Jolanta Karpinska, Andrea Erxleben, Patrick McArdle

**Affiliations:** a School of Chemistry, National University of Ireland, Galway, University Road, Galway, Ireland

## Abstract

The title compound, C_20_H_28_O_2_, is a steroid with strong anabolic properties. The present solvent-free form crystallizes with two mol­ecules per asymmetric unit. In the crystal, both mol­ecules are involved in the formation of O—H⋯O hydrogen-bonded chains which extend along the *b*-axis direction.

## Related literature

For examples of other compounds with unused hydrogen-bonding capacity, see: Bhatt *et al.* (2006 ▶); Lewis *et al.* (2005 ▶); Desiraju *et al.* (2002 ▶). For related structures of other anabolic steroids, see: Verma *et al.* (2006 ▶). For related structures of steroid compounds with non-hydrogen-bonded OH or C=O motifs, see: Karpinska *et al.* (2011 ▶); Danaci *et al.* (1988 ▶); Chakrabarti *et al.* (1981 ▶); McPhail *et al.* (1977 ▶); Delettré *et al.* (1975 ▶). For applications of methandrostenolone, see: Druzhinina *et al.* (2008 ▶). For a previously reported mono hydrate (with no unused hydrogen-bonding capacity), see: Duax *et al.* (1982 ▶).
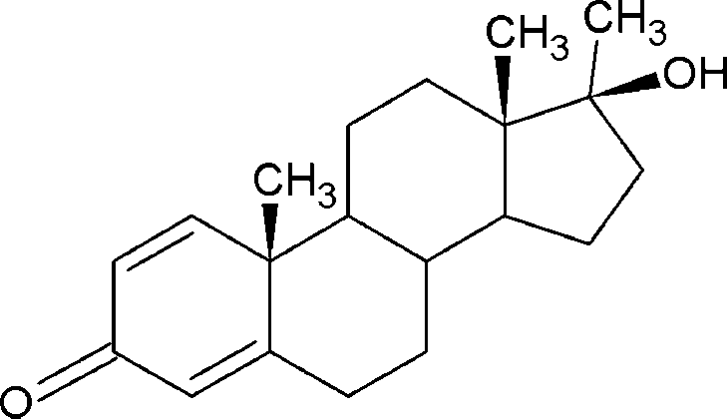


## Experimental

### 

#### Crystal data

C_20_H_28_O_2_
*M* *_r_* = 300.42
Monoclinic, 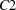

*a* = 28.317 (2) Å
*b* = 9.4539 (5) Å
*c* = 13.7684 (10) Å
β = 111.017 (9)°
*V* = 3440.7 (4) Å^3^
*Z* = 8
Mo *K*α radiation
μ = 0.07 mm^−1^
*T* = 298 K
0.50 × 0.40 × 0.20 mm

#### Data collection

Oxford Diffraction Xcalibur Sapphire3 diffractometer
Absorption correction: multi-scan (CrysAlis171; Oxford Diffraction, 2010 ▶) *T*_min_ = 0.985, *T*_max_ = 1.000
7321 measured reflections
4697 independent reflections
2893 reflections with *I* > 2σ(*I*)
*R*_int_ = 0.034

#### Refinement

*R*[*F* ^2^ > 2σ(*F* ^2^)] = 0.072
*wR*(*F* ^2^) = 0.199
*S* = 1.03
4697 reflections
404 parameters
1 restraint
H-atom parameters constrained
Δρ_max_ = 0.65 e Å^−3^
Δρ_min_ = −0.26 e Å^−3^

### 

Data collection: *CrysAlis171* (Oxford Diffraction, 2010 ▶); cell refinement: *CrysAlis171*; data reduction: *CrysAlis171*; program(s) used to solve structure: *SHELXS97* (Sheldrick, 2008 ▶); program(s) used to refine structure: *SHELXL97* (Sheldrick, 2008 ▶); molecular graphics: *ORTEX* (McArdle, 1995 ▶); software used to prepare material for publication: *CIFTAB* (Sheldrick, 2008 ▶).

## Supplementary Material

Crystal structure: contains datablock(s) I, global. DOI: 10.1107/S1600536812049860/bg2491sup1.cif

Structure factors: contains datablock(s) I. DOI: 10.1107/S1600536812049860/bg2491Isup3.hkl

Additional supplementary materials:  crystallographic information; 3D view; checkCIF report

## Figures and Tables

**Table 1:** Hydrogen-bond geometry (Å, °)

*D*—H⋯*A*	*D*—H	H⋯*A*	*D*⋯*A*	*D*—H⋯*A*
O2—H2*O*2⋯O4^i^	0.82	2.00	2.808 (7)	167
O4—H4*O*4⋯O3^ii^	0.82	2.09	2.858 (7)	156

Symmetry codes: (i) 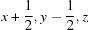; (ii) 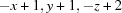.

**Fig. 1.**
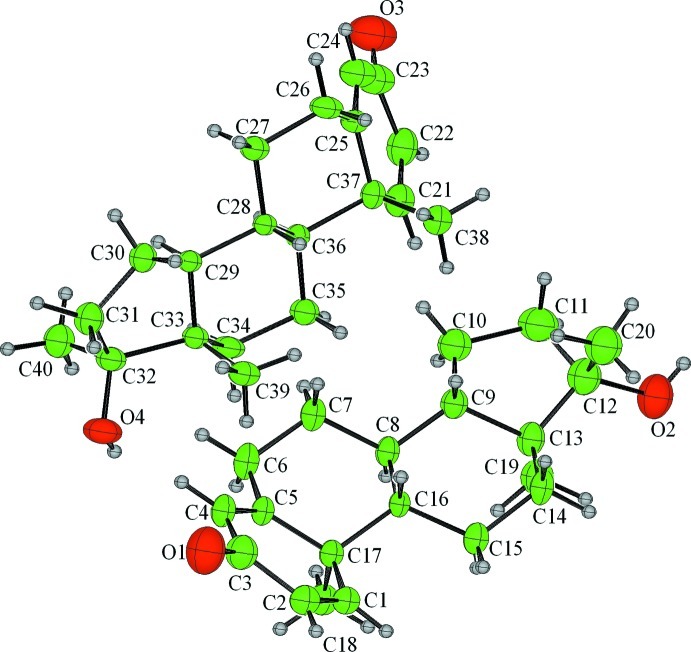
One-dimensional hydrogen bonded chains in the crystal structure of methandrostenolone.

**Fig. 2.**
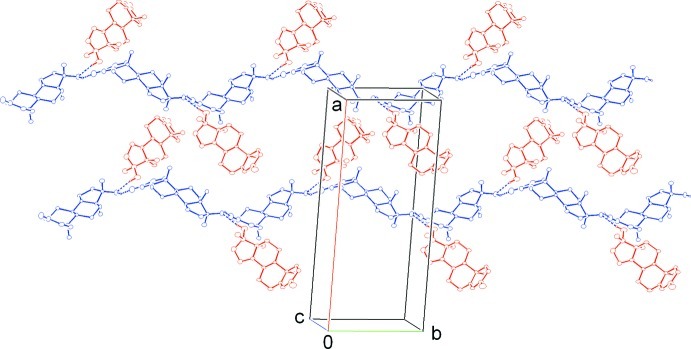
Packing diagram showing the 1D motifs along b with molecules coloured by symmetry equivalence.

## supplementary crystallographic information

### Comment

Methandrostenolone, commonly known as dianabol, is an anabolic steroid which is widely applied in medicine, particularly in surgery, endocrinology, therapeutics and paediatrics (Druzhinina *et al.*, 2008)). As a part of our studies on unused hydrogen bonding capacity in steroid compounds the crystal structure of the solvent free form has been determined using crystals grown by sublimation.

Methandrostenolone is another example of a steroid type compound which does not use all of its good proton donors and acceptors in hydrogen bonding formation. The previously reported mono hydrate has no unused hydrogen bonding capacity (Duax *et al.*, 1982). The OH and C=O groups occupy opposite ends of the molecule and both are used in the formation of one dimensional hydrogen bonded chains. The O4-H4O3..O3 (2.878 Å) interactions of one molecule form chains (blue molecules in Fig. 2), with the O2 of the hydroxyl groups of the other molecule acting only as donors to the basic chain in O2-H2O2..O4 (2.808 Å) interactions (red molecules in Fig. 2). The O1 atom of the carbonyl group of this second molecule is not involved in any hydrogen bonding interaction.

### Experimental

The title compound of high purity (>98.8%) was obtained from TCI Europe. Colourless crystals were grown by low temperature gradient sublimation in the vacuum.

### Refinement

All H atoms were included in the refinement in calculated positions [N—O = 0.82 Å, C—H(aromatic) = 0.93 Å, *C*—*H*(methylene) = 0.97 Å or *C*—*H*(methyl) = 0.96 Å] and allowed to ride on their parent atoms, with *U*_iso_(H) = 1.2*U*_eq_. The rather low "observed-to-unique" reflection ratio was due to extremly poor data quality.

### Crystal data

**Table:**

C_20_H_28_O_2_	*F*(000) = 1312
*M**_r_* = 300.42	*D*_x_ = 1.160 Mg m^−^^3^
Monoclinic, *C*2	Mo *K*α radiation, λ = 0.7107 Å
*a* = 28.317 (2) Å	Cell parameters from 1944 reflections
*b* = 9.4539 (5) Å	θ = 2.9–29.2°
*c* = 13.7684 (10) Å	µ = 0.07 mm^−^^1^
β = 111.017 (9)°	*T* = 298 K
*V* = 3440.7 (4) Å^3^	Parallelepiped, colourless
*Z* = 8	0.50 × 0.40 × 0.20 mm

### Data collection

**Table:**

Oxford Diffraction Xcalibur Sapphire3 diffractometer	4697 independent reflections
Radiation source: Enhance (Mo) X-ray Source	2893 reflections with *I* > 2σ(*I*)
Graphite monochromator	*R*_int_ = 0.034
Detector resolution: 16.1048 pixels mm^-1^	θ_max_ = 25.4°, θ_min_ = 2.9°
ω scans	*h* = −34→21
Absorption correction: multi-scan (CrysAlis171; Oxford Diffraction, 2010)	*k* = −8→11
*T*_min_ = 0.985, *T*_max_ = 1.000	*l* = −16→18
7321 measured reflections	

### Refinement

**Table:**

Refinement on *F*^2^	Secondary atom site location: difference Fourier map
Least-squares matrix: full	Hydrogen site location: inferred from neighbouring sites
*R*[*F*^2^ > 2σ(*F*^2^)] = 0.072	H-atom parameters constrained
*wR*(*F*^2^) = 0.199	*w* = 1/[σ^2^(*F*_o_^2^) + (0.0874*P*)^2^ + 2.2675*P*] where *P* = (*F*_o_^2^ + 2*F*_c_^2^)/3
*S* = 1.03	(Δ/σ)_max_ < 0.001
4697 reflections	Δρ_max_ = 0.65 e Å^−^^3^
404 parameters	Δρ_min_ = −0.26 e Å^−^^3^
1 restraint	Extinction correction: *SHELXL97* (Sheldrick, 2008), Fc^*^=kFc[1+0.001xFc^2^λ^3^/sin(2θ)]^-1/4^
Primary atom site location: structure-invariant direct methods	Extinction coefficient: 0.0068 (9)

### Special details

**Table:**

Geometry. All e.s.d.'s (except the e.s.d. in the dihedral angle between two l.s. planes) are estimated using the full covariance matrix. The cell e.s.d.'s are taken into account individually in the estimation of e.s.d.'s in distances, angles and torsion angles; correlations between e.s.d.'s in cell parameters are only used when they are defined by crystal symmetry. An approximate (isotropic) treatment of cell e.s.d.'s is used for estimating e.s.d.'s involving l.s. planes.

### Fractional atomic coordinates and isotropic or equivalent isotropic displacement parameters (Å^2^)

**Table:**

	*x*	*y*	*z*	*U*_iso_*/*U*_eq_	
O1	0.7034 (2)	1.0649 (8)	−0.1279 (3)	0.147 (3)	
O2	0.91191 (19)	0.6512 (8)	0.5818 (3)	0.138 (2)	
H2O2	0.9258	0.5758	0.6046	0.180*	
C1	0.7739 (2)	1.1398 (7)	0.1374 (5)	0.0760 (17)	
H1	0.8022	1.1918	0.1767	0.099*	
C2	0.7592 (3)	1.1452 (7)	0.0348 (6)	0.088 (2)	
H2A	0.7769	1.2022	0.0046	0.115*	
C3	0.7155 (3)	1.0635 (9)	−0.0328 (5)	0.091 (2)	
C4	0.6873 (2)	0.9890 (7)	0.0178 (4)	0.0770 (18)	
H4	0.6583	0.9413	−0.0231	0.100*	
C5	0.70045 (18)	0.9839 (6)	0.1216 (4)	0.0621 (14)	
C6	0.6729 (2)	0.8922 (8)	0.1723 (5)	0.090 (2)	
H6A	0.6440	0.8490	0.1194	0.117*	
H6B	0.6606	0.9495	0.2167	0.117*	
C7	0.7077 (2)	0.7781 (8)	0.2364 (4)	0.0793 (18)	
H7A	0.7147	0.7114	0.1898	0.103*	
H7B	0.6904	0.7272	0.2751	0.103*	
C8	0.75730 (19)	0.8347 (6)	0.3120 (4)	0.0619 (15)	
H8	0.7501	0.8896	0.3655	0.081*	
C9	0.7937 (2)	0.7179 (7)	0.3653 (4)	0.0685 (15)	
H9	0.8012	0.6680	0.3100	0.089*	
C10	0.7765 (3)	0.6027 (8)	0.4256 (5)	0.101 (2)	
H10A	0.7585	0.6440	0.4667	0.132*	
H10B	0.7550	0.5330	0.3786	0.132*	
C11	0.8267 (3)	0.5370 (9)	0.4953 (5)	0.116 (3)	
H11A	0.8290	0.4401	0.4744	0.151*	
H11B	0.8288	0.5369	0.5671	0.151*	
C12	0.8709 (3)	0.6272 (9)	0.4841 (5)	0.101 (3)	
C13	0.8448 (2)	0.7699 (8)	0.4417 (4)	0.0765 (18)	
C14	0.8693 (2)	0.8604 (8)	0.3831 (4)	0.0811 (19)	
H14A	0.8769	0.8029	0.3322	0.105*	
H14B	0.9010	0.8981	0.4312	0.105*	
C15	0.8344 (2)	0.9834 (7)	0.3275 (4)	0.0780 (18)	
H15A	0.8303	1.0470	0.3792	0.101*	
H15B	0.8502	1.0361	0.2868	0.101*	
C16	0.78270 (17)	0.9333 (6)	0.2564 (4)	0.0521 (13)	
H16	0.7892	0.8740	0.2041	0.068*	
C17	0.74705 (19)	1.0534 (6)	0.1933 (4)	0.0609 (14)	
C18	0.7340 (2)	1.1561 (8)	0.2678 (5)	0.093 (2)	
H18A	0.7112	1.2276	0.2279	0.121*	
H18B	0.7645	1.1998	0.3136	0.121*	
H18C	0.7183	1.1044	0.3081	0.121*	
C19	0.8392 (3)	0.8526 (9)	0.5324 (4)	0.098 (2)	
H19A	0.8719	0.8843	0.5778	0.128*	
H19B	0.8246	0.7926	0.5704	0.128*	
H19C	0.8176	0.9329	0.5060	0.128*	
C20	0.8936 (3)	0.5518 (9)	0.4139 (5)	0.120 (3)	
H20A	0.9214	0.6062	0.4095	0.156*	
H20B	0.8683	0.5415	0.3457	0.156*	
H20C	0.9055	0.4601	0.4421	0.156*	
O4	0.47262 (19)	0.9195 (5)	0.6785 (4)	0.1069 (16)	
H4O4	0.4754	0.9614	0.7324	0.139*	
O3	0.5497 (3)	0.0511 (7)	1.1560 (6)	0.172 (3)	
C21	0.6004 (2)	0.3797 (9)	1.1075 (5)	0.093 (2)	
H21	0.6120	0.4643	1.1423	0.121*	
C22	0.5869 (3)	0.2739 (10)	1.1603 (5)	0.097 (2)	
H22	0.5911	0.2871	1.2299	0.126*	
C23	0.5658 (3)	0.1402 (9)	1.1088 (7)	0.116 (3)	
C24	0.5673 (3)	0.1263 (8)	1.0132 (6)	0.105 (3)	
H24	0.5577	0.0385	0.9819	0.137*	
C25	0.5807 (2)	0.2215 (6)	0.9574 (5)	0.0689 (16)	
C26	0.5755 (3)	0.1940 (7)	0.8476 (5)	0.0845 (18)	
H26A	0.5643	0.0975	0.8289	0.110*	
H26B	0.6081	0.2057	0.8401	0.110*	
C27	0.5373 (2)	0.2967 (6)	0.7756 (4)	0.0678 (15)	
H27A	0.5039	0.2745	0.7756	0.088*	
H27B	0.5367	0.2843	0.7052	0.088*	
C28	0.54964 (18)	0.4516 (5)	0.8080 (3)	0.0475 (12)	
H28	0.5808	0.4785	0.7971	0.062*	
C29	0.50704 (18)	0.5476 (5)	0.7438 (3)	0.0482 (11)	
H29	0.4773	0.5200	0.7601	0.063*	
C30	0.4902 (2)	0.5470 (7)	0.6264 (4)	0.0693 (15)	
H30A	0.4680	0.4676	0.5971	0.090*	
H30B	0.5191	0.5415	0.6045	0.090*	
C31	0.4621 (2)	0.6876 (7)	0.5924 (4)	0.0781 (18)	
H31A	0.4267	0.6705	0.5520	0.101*	
H31B	0.4767	0.7410	0.5499	0.101*	
C32	0.4675 (2)	0.7705 (6)	0.6926 (4)	0.0695 (15)	
C33	0.51633 (19)	0.7034 (5)	0.7731 (4)	0.0562 (13)	
C34	0.5242 (3)	0.7212 (6)	0.8868 (4)	0.0762 (17)	
H34A	0.4933	0.6968	0.8980	0.099*	
H34B	0.5319	0.8194	0.9065	0.099*	
C35	0.5670 (2)	0.6281 (6)	0.9545 (4)	0.0728 (16)	
H35A	0.5705	0.6393	1.0268	0.095*	
H35B	0.5985	0.6576	0.9476	0.095*	
C36	0.55717 (19)	0.4725 (5)	0.9237 (4)	0.0517 (12)	
H36	0.5249	0.4481	0.9307	0.067*	
C37	0.59779 (19)	0.3678 (7)	0.9965 (4)	0.0630 (15)	
C38	0.6512 (2)	0.3981 (7)	0.9955 (5)	0.0871 (19)	
H38A	0.6607	0.4935	1.0178	0.113*	
H38B	0.6512	0.3857	0.9263	0.113*	
H38C	0.6750	0.3338	1.0418	0.113*	
C39	0.5624 (2)	0.7593 (7)	0.7519 (5)	0.0883 (19)	
H39A	0.5671	0.8576	0.7701	0.115*	
H39B	0.5570	0.7481	0.6794	0.115*	
H39C	0.5919	0.7071	0.7928	0.115*	
C40	0.4201 (2)	0.7533 (8)	0.7211 (5)	0.094 (2)	
H40A	0.4236	0.8097	0.7813	0.122*	
H40B	0.4163	0.6557	0.7361	0.122*	
H40C	0.3909	0.7837	0.6639	0.122*	

### Atomic displacement parameters (Å^2^)

**Table:**

	*U*^11^	*U*^22^	*U*^33^	*U*^12^	*U*^13^	*U*^23^
O1	0.161 (5)	0.225 (7)	0.065 (3)	0.046 (5)	0.054 (3)	0.040 (4)
O2	0.128 (4)	0.197 (6)	0.066 (3)	0.054 (4)	0.007 (3)	0.033 (4)
C1	0.073 (4)	0.074 (4)	0.086 (4)	0.007 (3)	0.035 (3)	0.022 (4)
C2	0.089 (4)	0.086 (5)	0.104 (5)	0.026 (4)	0.052 (4)	0.046 (4)
C3	0.104 (5)	0.117 (6)	0.066 (4)	0.044 (5)	0.047 (4)	0.035 (4)
C4	0.058 (3)	0.106 (5)	0.059 (3)	0.016 (3)	0.011 (3)	0.004 (4)
C5	0.049 (3)	0.082 (4)	0.056 (3)	0.012 (3)	0.020 (3)	0.008 (3)
C6	0.058 (3)	0.134 (6)	0.083 (4)	−0.003 (4)	0.030 (3)	0.013 (5)
C7	0.060 (3)	0.110 (5)	0.072 (4)	−0.008 (4)	0.028 (3)	0.017 (4)
C8	0.056 (3)	0.089 (4)	0.045 (3)	0.007 (3)	0.024 (3)	0.006 (3)
C9	0.074 (4)	0.088 (4)	0.052 (3)	0.015 (3)	0.034 (3)	0.014 (3)
C10	0.114 (5)	0.103 (5)	0.097 (5)	0.008 (5)	0.050 (4)	0.035 (5)
C11	0.168 (8)	0.113 (6)	0.088 (5)	0.049 (6)	0.069 (6)	0.039 (5)
C12	0.116 (5)	0.133 (7)	0.055 (4)	0.057 (5)	0.032 (4)	0.029 (4)
C13	0.077 (4)	0.113 (5)	0.039 (3)	0.029 (4)	0.020 (3)	0.009 (3)
C14	0.053 (3)	0.124 (6)	0.060 (3)	0.006 (4)	0.011 (3)	0.011 (4)
C15	0.062 (3)	0.100 (5)	0.068 (4)	0.002 (3)	0.019 (3)	0.014 (4)
C16	0.043 (2)	0.068 (3)	0.046 (3)	0.005 (2)	0.018 (2)	0.007 (3)
C17	0.057 (3)	0.074 (4)	0.057 (3)	0.010 (3)	0.027 (3)	0.009 (3)
C18	0.095 (4)	0.096 (5)	0.090 (4)	0.027 (4)	0.036 (4)	−0.001 (4)
C19	0.109 (5)	0.123 (6)	0.061 (4)	0.027 (5)	0.027 (4)	−0.003 (4)
C20	0.141 (6)	0.145 (7)	0.080 (4)	0.064 (6)	0.048 (5)	0.020 (5)
O4	0.157 (4)	0.062 (3)	0.095 (3)	0.024 (3)	0.037 (3)	0.025 (3)
O3	0.244 (7)	0.123 (5)	0.217 (7)	0.044 (5)	0.166 (6)	0.085 (5)
C21	0.087 (4)	0.109 (6)	0.085 (4)	0.040 (4)	0.033 (4)	0.008 (5)
C22	0.098 (5)	0.144 (7)	0.058 (4)	0.040 (5)	0.040 (4)	0.036 (5)
C23	0.156 (7)	0.089 (6)	0.141 (7)	0.056 (6)	0.100 (7)	0.053 (6)
C24	0.132 (6)	0.077 (5)	0.146 (7)	0.040 (5)	0.096 (6)	0.049 (5)
C25	0.077 (4)	0.051 (3)	0.096 (4)	0.017 (3)	0.052 (4)	0.024 (3)
C26	0.114 (5)	0.047 (3)	0.100 (5)	0.007 (4)	0.049 (4)	0.003 (4)
C27	0.087 (4)	0.057 (4)	0.069 (4)	0.005 (3)	0.040 (3)	−0.008 (3)
C28	0.058 (3)	0.044 (3)	0.049 (3)	0.002 (2)	0.029 (2)	−0.002 (2)
C29	0.061 (3)	0.045 (3)	0.043 (2)	0.001 (3)	0.023 (2)	−0.002 (2)
C30	0.078 (4)	0.084 (4)	0.044 (3)	0.013 (3)	0.020 (3)	0.003 (3)
C31	0.090 (4)	0.104 (5)	0.040 (3)	0.033 (4)	0.024 (3)	0.013 (3)
C32	0.092 (4)	0.056 (3)	0.061 (3)	0.021 (3)	0.028 (3)	0.015 (3)
C33	0.070 (3)	0.049 (3)	0.053 (3)	0.008 (3)	0.025 (3)	0.010 (3)
C34	0.112 (5)	0.044 (3)	0.066 (4)	0.017 (3)	0.024 (4)	−0.007 (3)
C35	0.089 (4)	0.067 (4)	0.055 (3)	0.007 (4)	0.017 (3)	−0.012 (3)
C36	0.060 (3)	0.052 (3)	0.047 (3)	0.007 (2)	0.024 (2)	−0.004 (2)
C37	0.060 (3)	0.081 (4)	0.054 (3)	0.021 (3)	0.027 (3)	0.013 (3)
C38	0.080 (4)	0.086 (5)	0.101 (5)	0.011 (4)	0.039 (4)	0.007 (4)
C39	0.100 (5)	0.058 (4)	0.107 (5)	−0.014 (4)	0.037 (4)	0.015 (4)
C40	0.095 (4)	0.111 (5)	0.085 (4)	0.047 (4)	0.042 (4)	0.014 (4)

### Geometric parameters (Å, º)

**Table:**

O1—C3	1.228 (7)	O4—C32	1.436 (7)
O2—C12	1.445 (8)	O4—H4O4	0.8200
O2—H2O2	0.8200	O3—C23	1.246 (8)
C1—C2	1.322 (9)	C21—C22	1.370 (10)
C1—C17	1.504 (7)	C21—C37	1.507 (7)
C1—H1	0.9300	C21—H21	0.9300
C2—C3	1.472 (9)	C22—C23	1.468 (12)
C2—H2A	0.9300	C22—H22	0.9300
C3—C4	1.420 (9)	C23—C24	1.339 (10)
C4—C5	1.343 (7)	C24—C25	1.325 (8)
C4—H4	0.9300	C24—H24	0.9300
C5—C17	1.488 (7)	C25—C26	1.488 (8)
C5—C6	1.495 (8)	C25—C37	1.499 (9)
C6—C7	1.513 (9)	C26—C27	1.526 (8)
C6—H6A	0.9700	C26—H26A	0.9700
C6—H6B	0.9700	C26—H26B	0.9700
C7—C8	1.515 (8)	C27—C28	1.534 (7)
C7—H7A	0.9700	C27—H27A	0.9700
C7—H7B	0.9700	C27—H27B	0.9700
C8—C9	1.510 (7)	C28—C29	1.515 (6)
C8—C16	1.539 (6)	C28—C36	1.542 (6)
C8—H8	0.9800	C28—H28	0.9800
C9—C13	1.532 (8)	C29—C30	1.513 (6)
C9—C10	1.551 (8)	C29—C33	1.525 (7)
C9—H9	0.9800	C29—H29	0.9800
C10—C11	1.531 (9)	C30—C31	1.534 (8)
C10—H10A	0.9700	C30—H30A	0.9700
C10—H10B	0.9700	C30—H30B	0.9700
C11—C12	1.568 (11)	C31—C32	1.545 (7)
C11—H11A	0.9700	C31—H31A	0.9700
C11—H11B	0.9700	C31—H31B	0.9700
C12—C20	1.516 (9)	C32—C40	1.537 (8)
C12—C13	1.549 (10)	C32—C33	1.564 (7)
C13—C14	1.507 (8)	C33—C34	1.509 (7)
C13—C19	1.529 (8)	C33—C39	1.530 (7)
C14—C15	1.541 (9)	C34—C35	1.518 (7)
C14—H14A	0.9700	C34—H34A	0.9700
C14—H14B	0.9700	C34—H34B	0.9700
C15—C16	1.516 (7)	C35—C36	1.529 (7)
C15—H15A	0.9700	C35—H35A	0.9700
C15—H15B	0.9700	C35—H35B	0.9700
C16—C17	1.560 (7)	C36—C37	1.575 (7)
C16—H16	0.9800	C36—H36	0.9800
C17—C18	1.550 (8)	C37—C38	1.545 (8)
C18—H18A	0.9600	C38—H38A	0.9600
C18—H18B	0.9600	C38—H38B	0.9600
C18—H18C	0.9600	C38—H38C	0.9600
C19—H19A	0.9600	C39—H39A	0.9600
C19—H19B	0.9600	C39—H39B	0.9600
C19—H19C	0.9600	C39—H39C	0.9600
C20—H20A	0.9600	C40—H40A	0.9600
C20—H20B	0.9600	C40—H40B	0.9600
C20—H20C	0.9600	C40—H40C	0.9600
			
C12—O2—H2O2	109.5	C32—O4—H4O4	109.5
C2—C1—C17	123.0 (6)	C22—C21—C37	124.2 (7)
C2—C1—H1	118.5	C22—C21—H21	117.9
C17—C1—H1	118.5	C37—C21—H21	117.9
C1—C2—C3	121.8 (6)	C21—C22—C23	121.1 (6)
C1—C2—H2A	119.1	C21—C22—H22	119.5
C3—C2—H2A	119.1	C23—C22—H22	119.5
O1—C3—C4	122.5 (8)	O3—C23—C24	126.4 (10)
O1—C3—C2	121.2 (7)	O3—C23—C22	119.5 (8)
C4—C3—C2	116.2 (5)	C24—C23—C22	114.1 (7)
C5—C4—C3	123.4 (6)	C25—C24—C23	128.4 (8)
C5—C4—H4	118.3	C25—C24—H24	115.8
C3—C4—H4	118.3	C23—C24—H24	115.8
C4—C5—C17	122.1 (5)	C24—C25—C26	121.7 (6)
C4—C5—C6	121.7 (6)	C24—C25—C37	122.8 (6)
C17—C5—C6	115.7 (4)	C26—C25—C37	115.3 (5)
C5—C6—C7	110.3 (4)	C25—C26—C27	109.8 (5)
C5—C6—H6A	109.6	C25—C26—H26A	109.7
C7—C6—H6A	109.6	C27—C26—H26A	109.7
C5—C6—H6B	109.6	C25—C26—H26B	109.7
C7—C6—H6B	109.6	C27—C26—H26B	109.7
H6A—C6—H6B	108.1	H26A—C26—H26B	108.2
C6—C7—C8	113.5 (6)	C26—C27—C28	112.5 (5)
C6—C7—H7A	108.9	C26—C27—H27A	109.1
C8—C7—H7A	108.9	C28—C27—H27A	109.1
C6—C7—H7B	108.9	C26—C27—H27B	109.1
C8—C7—H7B	108.9	C28—C27—H27B	109.1
H7A—C7—H7B	107.7	H27A—C27—H27B	107.8
C9—C8—C7	112.3 (5)	C29—C28—C27	110.5 (4)
C9—C8—C16	108.9 (4)	C29—C28—C36	108.2 (3)
C7—C8—C16	110.8 (4)	C27—C28—C36	110.9 (4)
C9—C8—H8	108.2	C29—C28—H28	109.1
C7—C8—H8	108.2	C27—C28—H28	109.1
C16—C8—H8	108.2	C36—C28—H28	109.1
C8—C9—C13	114.3 (5)	C30—C29—C28	120.1 (4)
C8—C9—C10	118.8 (4)	C30—C29—C33	104.1 (4)
C13—C9—C10	105.0 (5)	C28—C29—C33	113.5 (4)
C8—C9—H9	105.9	C30—C29—H29	106.1
C13—C9—H9	105.9	C28—C29—H29	106.1
C10—C9—H9	105.9	C33—C29—H29	106.1
C11—C10—C9	102.8 (5)	C29—C30—C31	104.7 (4)
C11—C10—H10A	111.2	C29—C30—H30A	110.8
C9—C10—H10A	111.2	C31—C30—H30A	110.8
C11—C10—H10B	111.2	C29—C30—H30B	110.8
C9—C10—H10B	111.2	C31—C30—H30B	110.8
H10A—C10—H10B	109.1	H30A—C30—H30B	108.9
C10—C11—C12	108.2 (6)	C30—C31—C32	107.0 (4)
C10—C11—H11A	110.1	C30—C31—H31A	110.3
C12—C11—H11A	110.1	C32—C31—H31A	110.3
C10—C11—H11B	110.1	C30—C31—H31B	110.3
C12—C11—H11B	110.1	C32—C31—H31B	110.3
H11A—C11—H11B	108.4	H31A—C31—H31B	108.6
O2—C12—C20	106.6 (6)	O4—C32—C40	106.2 (5)
O2—C12—C13	109.3 (7)	O4—C32—C31	111.1 (5)
C20—C12—C13	115.0 (5)	C40—C32—C31	111.0 (5)
O2—C12—C11	113.4 (5)	O4—C32—C33	112.8 (5)
C20—C12—C11	110.1 (7)	C40—C32—C33	114.0 (4)
C13—C12—C11	102.7 (5)	C31—C32—C33	101.9 (4)
C14—C13—C19	110.5 (6)	C34—C33—C29	109.3 (4)
C14—C13—C9	108.5 (4)	C34—C33—C39	110.0 (5)
C19—C13—C9	112.1 (5)	C29—C33—C39	111.2 (4)
C14—C13—C12	116.5 (5)	C34—C33—C32	117.0 (4)
C19—C13—C12	108.2 (5)	C29—C33—C32	100.3 (4)
C9—C13—C12	100.7 (6)	C39—C33—C32	108.8 (4)
C13—C14—C15	110.9 (4)	C33—C34—C35	111.1 (4)
C13—C14—H14A	109.5	C33—C34—H34A	109.4
C15—C14—H14A	109.5	C35—C34—H34A	109.4
C13—C14—H14B	109.5	C33—C34—H34B	109.4
C15—C14—H14B	109.5	C35—C34—H34B	109.4
H14A—C14—H14B	108.0	H34A—C34—H34B	108.0
C16—C15—C14	112.6 (5)	C34—C35—C36	111.2 (4)
C16—C15—H15A	109.1	C34—C35—H35A	109.4
C14—C15—H15A	109.1	C36—C35—H35A	109.4
C16—C15—H15B	109.1	C34—C35—H35B	109.4
C14—C15—H15B	109.1	C36—C35—H35B	109.4
H15A—C15—H15B	107.8	H35A—C35—H35B	108.0
C15—C16—C8	112.6 (4)	C35—C36—C28	110.8 (4)
C15—C16—C17	114.5 (5)	C35—C36—C37	114.1 (4)
C8—C16—C17	113.2 (4)	C28—C36—C37	112.1 (4)
C15—C16—H16	105.2	C35—C36—H36	106.4
C8—C16—H16	105.2	C28—C36—H36	106.4
C17—C16—H16	105.2	C37—C36—H36	106.4
C5—C17—C1	113.0 (4)	C25—C37—C21	109.0 (5)
C5—C17—C18	110.6 (4)	C25—C37—C38	111.0 (5)
C1—C17—C18	106.4 (5)	C21—C37—C38	107.6 (5)
C5—C17—C16	106.9 (4)	C25—C37—C36	106.5 (4)
C1—C17—C16	109.6 (4)	C21—C37—C36	111.0 (4)
C18—C17—C16	110.3 (4)	C38—C37—C36	111.8 (4)
C17—C18—H18A	109.5	C37—C38—H38A	109.5
C17—C18—H18B	109.5	C37—C38—H38B	109.5
H18A—C18—H18B	109.5	H38A—C38—H38B	109.5
C17—C18—H18C	109.5	C37—C38—H38C	109.5
H18A—C18—H18C	109.5	H38A—C38—H38C	109.5
H18B—C18—H18C	109.5	H38B—C38—H38C	109.5
C13—C19—H19A	109.5	C33—C39—H39A	109.5
C13—C19—H19B	109.5	C33—C39—H39B	109.5
H19A—C19—H19B	109.5	H39A—C39—H39B	109.5
C13—C19—H19C	109.5	C33—C39—H39C	109.5
H19A—C19—H19C	109.5	H39A—C39—H39C	109.5
H19B—C19—H19C	109.5	H39B—C39—H39C	109.5
C12—C20—H20A	109.5	C32—C40—H40A	109.5
C12—C20—H20B	109.5	C32—C40—H40B	109.5
H20A—C20—H20B	109.5	H40A—C40—H40B	109.5
C12—C20—H20C	109.5	C32—C40—H40C	109.5
H20A—C20—H20C	109.5	H40A—C40—H40C	109.5
H20B—C20—H20C	109.5	H40B—C40—H40C	109.5
			
C17—C1—C2—C3	−1.4 (9)	C37—C21—C22—C23	−3.2 (10)
C1—C2—C3—O1	−177.0 (7)	C21—C22—C23—O3	−173.6 (7)
C1—C2—C3—C4	5.6 (9)	C21—C22—C23—C24	7.9 (10)
O1—C3—C4—C5	178.6 (7)	O3—C23—C24—C25	174.8 (7)
C2—C3—C4—C5	−4.0 (9)	C22—C23—C24—C25	−6.9 (12)
C3—C4—C5—C17	−1.8 (9)	C23—C24—C25—C26	−173.8 (7)
C3—C4—C5—C6	−173.5 (6)	C23—C24—C25—C37	0.7 (11)
C4—C5—C6—C7	115.1 (6)	C24—C25—C26—C27	115.7 (6)
C17—C5—C6—C7	−57.1 (7)	C37—C25—C26—C27	−59.2 (6)
C5—C6—C7—C8	51.8 (7)	C25—C26—C27—C28	53.1 (6)
C6—C7—C8—C9	−172.9 (4)	C26—C27—C28—C29	−172.1 (4)
C6—C7—C8—C16	−50.8 (6)	C26—C27—C28—C36	−52.2 (6)
C7—C8—C9—C13	179.3 (4)	C27—C28—C29—C30	−56.9 (6)
C16—C8—C9—C13	56.1 (5)	C36—C28—C29—C30	−178.5 (4)
C7—C8—C9—C10	−55.8 (6)	C27—C28—C29—C33	179.0 (4)
C16—C8—C9—C10	−179.0 (5)	C36—C28—C29—C33	57.5 (5)
C8—C9—C10—C11	−162.1 (5)	C28—C29—C30—C31	−160.2 (5)
C13—C9—C10—C11	−32.8 (6)	C33—C29—C30—C31	−31.8 (5)
C9—C10—C11—C12	7.1 (7)	C29—C30—C31—C32	4.6 (6)
C10—C11—C12—O2	138.2 (6)	C30—C31—C32—O4	143.7 (5)
C10—C11—C12—C20	−102.5 (6)	C30—C31—C32—C40	−98.4 (6)
C10—C11—C12—C13	20.5 (7)	C30—C31—C32—C33	23.4 (6)
C8—C9—C13—C14	−59.4 (6)	C30—C29—C33—C34	169.8 (4)
C10—C9—C13—C14	168.6 (5)	C28—C29—C33—C34	−57.9 (5)
C8—C9—C13—C19	62.9 (6)	C30—C29—C33—C39	−68.7 (5)
C10—C9—C13—C19	−69.0 (7)	C28—C29—C33—C39	63.6 (5)
C8—C9—C13—C12	177.7 (4)	C30—C29—C33—C32	46.2 (4)
C10—C9—C13—C12	45.8 (5)	C28—C29—C33—C32	178.5 (4)
O2—C12—C13—C14	82.6 (6)	O4—C32—C33—C34	80.9 (6)
C20—C12—C13—C14	−37.2 (9)	C40—C32—C33—C34	−40.3 (7)
C11—C12—C13—C14	−156.7 (5)	C31—C32—C33—C34	−160.0 (5)
O2—C12—C13—C19	−42.6 (7)	O4—C32—C33—C29	−161.1 (4)
C20—C12—C13—C19	−162.4 (7)	C40—C32—C33—C29	77.7 (6)
C11—C12—C13—C19	78.1 (6)	C31—C32—C33—C29	−42.0 (5)
O2—C12—C13—C9	−160.3 (5)	O4—C32—C33—C39	−44.4 (6)
C20—C12—C13—C9	79.9 (7)	C40—C32—C33—C39	−165.6 (5)
C11—C12—C13—C9	−39.7 (6)	C31—C32—C33—C39	74.7 (5)
C19—C13—C14—C15	−67.1 (6)	C29—C33—C34—C35	56.0 (6)
C9—C13—C14—C15	56.3 (7)	C39—C33—C34—C35	−66.2 (6)
C12—C13—C14—C15	168.9 (6)	C32—C33—C34—C35	169.1 (5)
C13—C14—C15—C16	−55.1 (6)	C33—C34—C35—C36	−57.3 (7)
C14—C15—C16—C8	52.4 (6)	C34—C35—C36—C28	57.2 (6)
C14—C15—C16—C17	−176.5 (4)	C34—C35—C36—C37	−175.2 (4)
C9—C8—C16—C15	−51.5 (6)	C29—C28—C36—C35	−56.0 (5)
C7—C8—C16—C15	−175.6 (5)	C27—C28—C36—C35	−177.4 (4)
C9—C8—C16—C17	176.7 (4)	C29—C28—C36—C37	175.3 (4)
C7—C8—C16—C17	52.7 (6)	C27—C28—C36—C37	53.9 (5)
C4—C5—C17—C1	5.8 (8)	C24—C25—C37—C21	4.3 (7)
C6—C5—C17—C1	178.0 (5)	C26—C25—C37—C21	179.1 (5)
C4—C5—C17—C18	125.0 (6)	C24—C25—C37—C38	122.6 (6)
C6—C5—C17—C18	−62.8 (7)	C26—C25—C37—C38	−62.6 (6)
C4—C5—C17—C16	−114.9 (6)	C24—C25—C37—C36	−115.5 (6)
C6—C5—C17—C16	57.3 (6)	C26—C25—C37—C36	59.4 (6)
C2—C1—C17—C5	−4.2 (8)	C22—C21—C37—C25	−2.8 (8)
C2—C1—C17—C18	−125.7 (6)	C22—C21—C37—C38	−123.2 (6)
C2—C1—C17—C16	115.0 (6)	C22—C21—C37—C36	114.2 (6)
C15—C16—C17—C5	174.9 (4)	C35—C36—C37—C25	177.5 (4)
C8—C16—C17—C5	−54.3 (5)	C28—C36—C37—C25	−55.5 (5)
C15—C16—C17—C1	52.1 (6)	C35—C36—C37—C21	59.1 (6)
C8—C16—C17—C1	−177.1 (5)	C28—C36—C37—C21	−174.0 (5)
C15—C16—C17—C18	−64.8 (6)	C35—C36—C37—C38	−61.1 (6)
C8—C16—C17—C18	66.0 (6)	C28—C36—C37—C38	65.9 (6)

### Hydrogen-bond geometry (Å, º)

**Table:**

*D*—H···*A*	*D*—H	H···*A*	*D*···*A*	*D*—H···*A*
O2—H2*O*2···O4^i^	0.82	2.00	2.808 (7)	167
O4—H4*O*4···O3^ii^	0.82	2.09	2.858 (7)	156

Symmetry codes: (i) *x*+1/2, *y*−1/2, *z*; (ii) −*x*+1, *y*+1, −*z*+2.
